# The London University

**Published:** 1896-05-30

**Authors:** 


### THE LONDON UNIVERSITY.

The controversy about the constitution and reorganisation of the London University, after smouldering for a few months, has cropped up again *apropos* of an election to the Council. The University of London may not be strong in orators, but, at least, it is prolific in scribes, and again we are threatened with long expositions of the different aspects of the question with all their irreconcilable differences.

But all these things have been said before. There is not a new suggestion to be made. The matter has been under almost constant discussion for more than fifteen years. A responsible Royal Commission has heard all sorts of evidence and has investigated every side of the problem. This Royal Commission has issued a very definite report, containing recommendations, the aim of which is unmistakable and the efficacy of which is undoubted; and the principle contained in these recommendations has received the approval of the Senate and of Convocation—the two responsible bodies within the University itself. The time for discussion, then, and for legitimate obstruction has surely long since gone by; action is what is now required, and nothing should be allowed to stand in the way of the appointment by Parliament of a Statutory Commission, as recommended by the recent Royal Commission, with powers to settle the necessary arrangements and regulations.

It is, no doubt, true that a large and, in some sense, an important section of Convocation takes strong objection to the proposed scheme. But it must be borne in mind that for years and years back the question, although not the precise proposal which is now before them, has been discussed, and that throughout all these endless discussions much the same class of objections have been put forward.

The differences, in fact, are radical, and, so far as existing graduates are concerned, the question appeals personally to many of them in a very different manner to that in which it does to those who regard it solely as it concerns the public welfare, and more especially the welfare of residents in London and students at London colleges. All the more necessary is it, then, that those on whom the ultimate decision will lie—that is, the Legislature—should harden their hearts and determine not to be too much influenced by people within the University, a body which, after all these years of discussion, has failed to make up its mind.

It seems to us that far too much has been made of the assumption that the London University is a purely examining body. On that assumption hangs the saying that the London University is a University not for London, but for the world. In a sense and in regard to certain degrees this is true, and no doubt one of the points on which this University may most legitimately pride itself (a form of self-praise by-the-bye in which its members are by no means backward) is that it has given large and effective encouragement to good work among students who have been cut off from colleges and deprived of academic advantages. But at the same time it must be remembered that to do this was no part of its original function. At first it admitted to its degrees only those who were students in some affiliated college or institution, and it is somewhat curious now to recall the fact that when that great change was made which so largely separated the Universities from teaching institutions, and according to some raised it to its present high position, the then graduates were even more opposed to the change than some of the present graduates are now to change in the opposite direction.

But the University never was, even in its latter days, as so many people would have us believe, a purely examining body. Its degrees in medicine have always been strictly confined to students at certain colleges and to students who have gone through certain accurately-defined courses of instruction by recognised teachers. Modern proposals, then, to make the London University fulfil the functions of a teaching University for London have about them nothing subversive of its original constitution. In any case it is high time that a definite end were made of the present condition of doubt and uncertainty. It is entirely derogatory to London, with its vast educational opportunities, that it should have no degree-giving University suitable for its own wants. Scotland, with a smaller population, has four Universities; Ireland with a slightly larger one has two; London has to share with the rest of the world in its one university, whose regulations are drawn to accommodate the outsiders rather than the Londoners. This is clearly absurd, and it is high time that in any reform the educational wants of Londoners should be considered rather than the fancied rights of those who are already members of the so-called London University.

